# Supplementary material for: Risk Factors for Mortality in Cardiac Implantable Electronic Device (CIED) Infections: A Systematic Review and Meta-Analysis
Source: J Clin Med. 2022 May 29;11(11):3063. doi: 10.3390/jcm11113063 (PMC9181812; doi:10.3390/jcm11113063)

Supplementary Figures

Supplemental Figure S1: Funnel plot for *Staphylococcus aureus* compared with non-*Staphylococcus aureus* infection as a risk factor for mortality

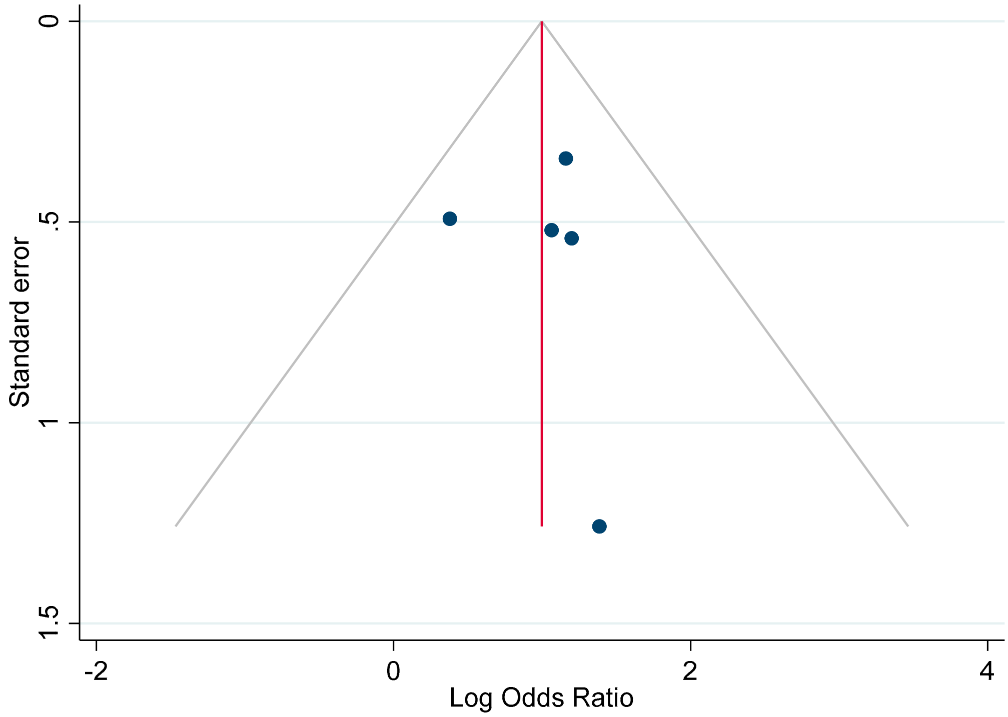

Supplemental Figure S2: Funnel plot for presence of embolism compared with no embolism as a risk factor for mortality

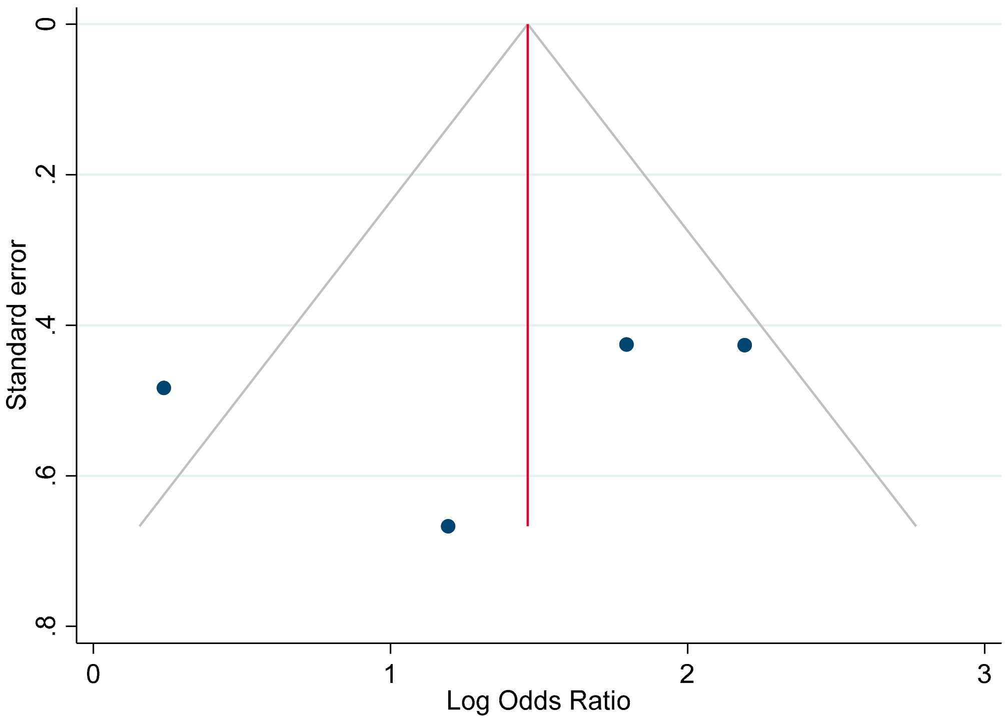

Supplemental Figure S3: Funnel plot for heart failure compared with no heart failure as a risk factor for mortality

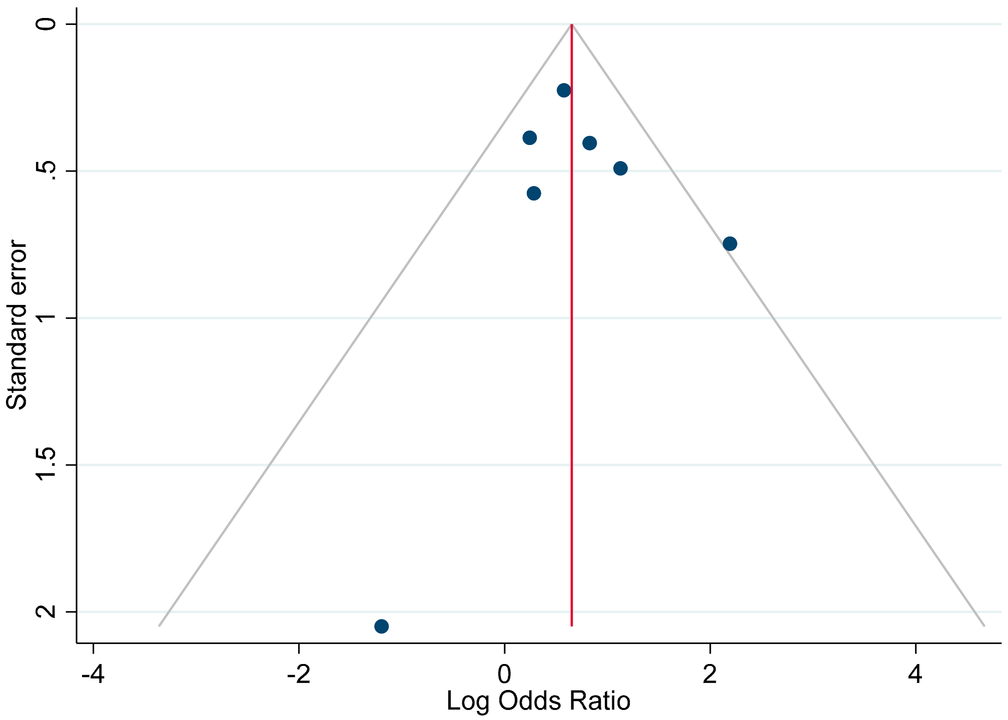

Supplemental Figure S4: Funnel plot for the males compared with females as a risk factor for mortality

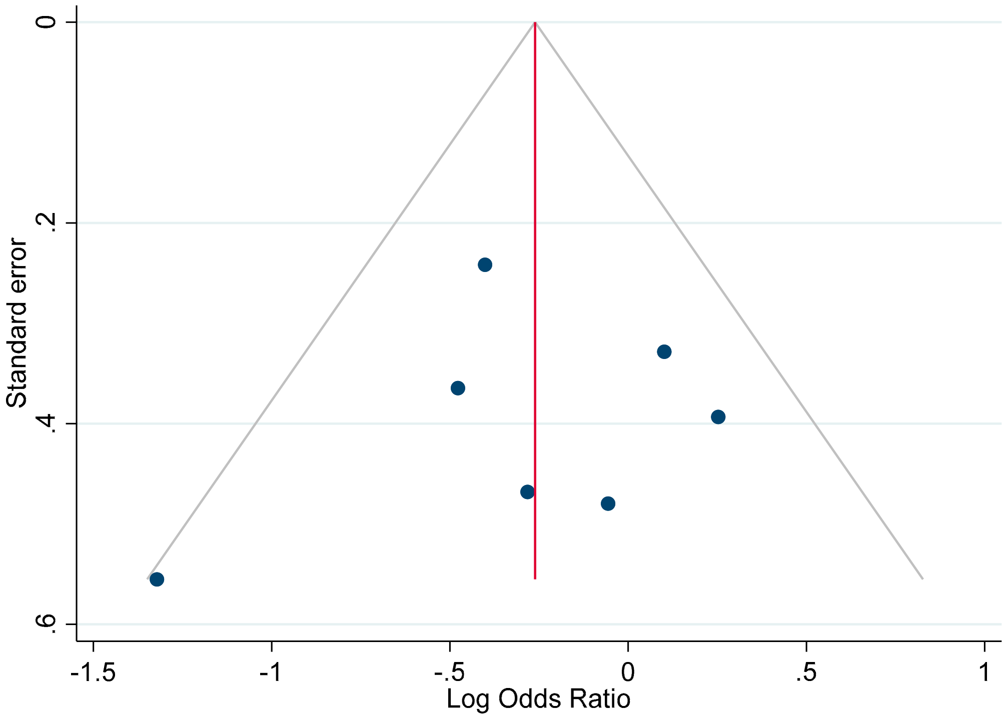

Supplemental Figure S5: Funnel plot for presence of diabetes mellitus as risk factor for mortality

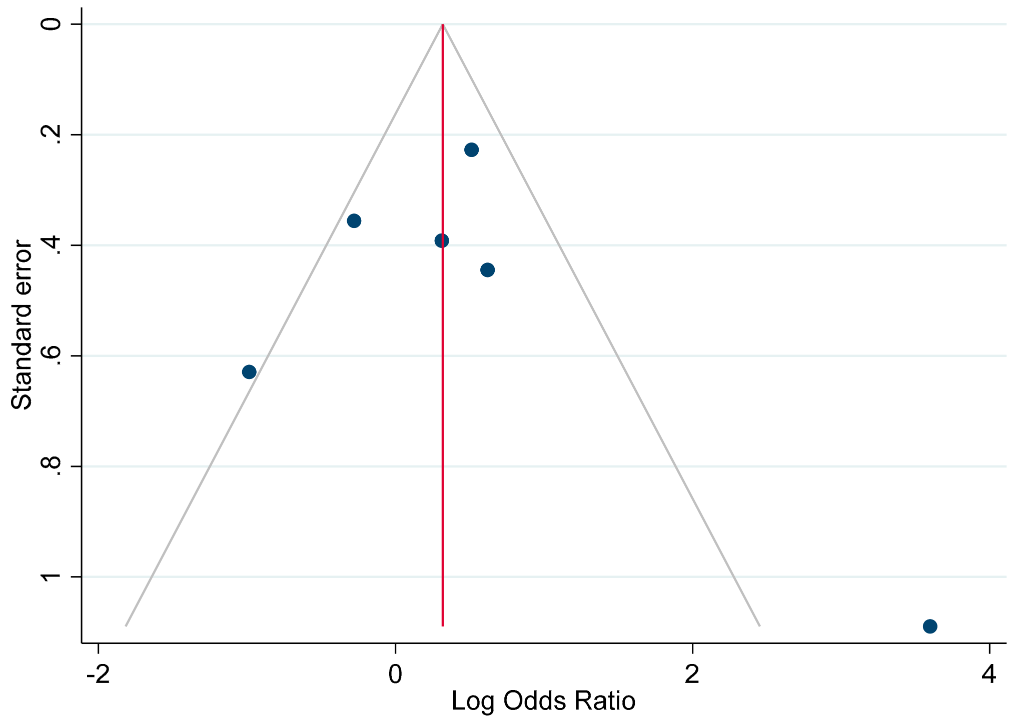

Supplemental Figure S6: Funnel plot for implantable cardioverter defibrillator compared with anti-bradycardiac CIED (pacemaker) as a risk factor for mortality

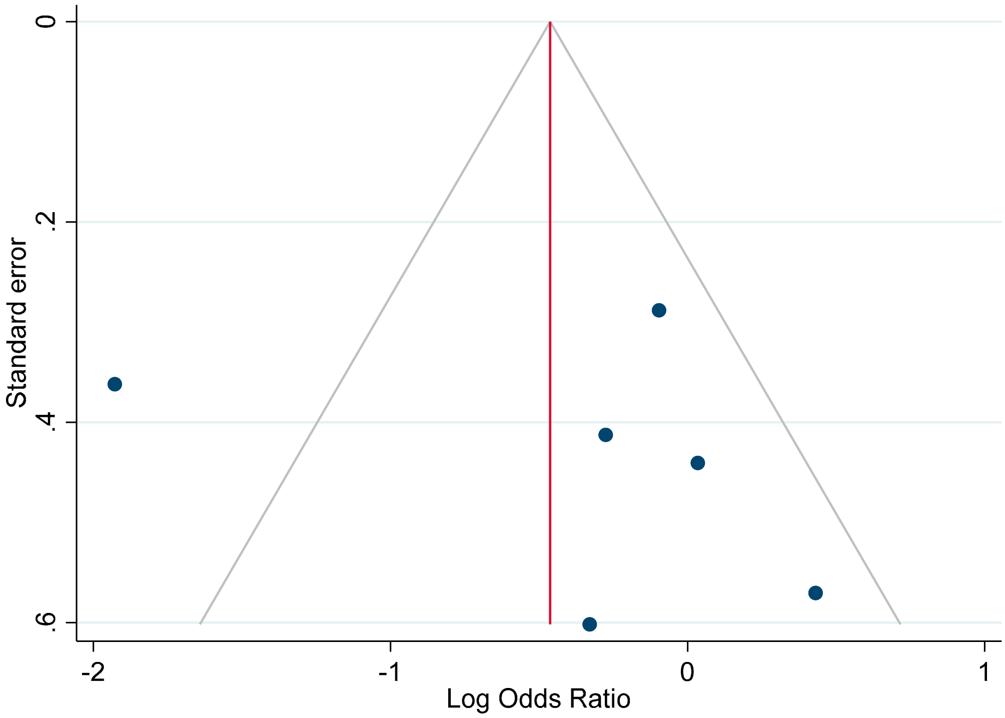

Supplemental Figure S7: Funnel plot for surgery compared with conservative medical therapy as a risk factor for mortality

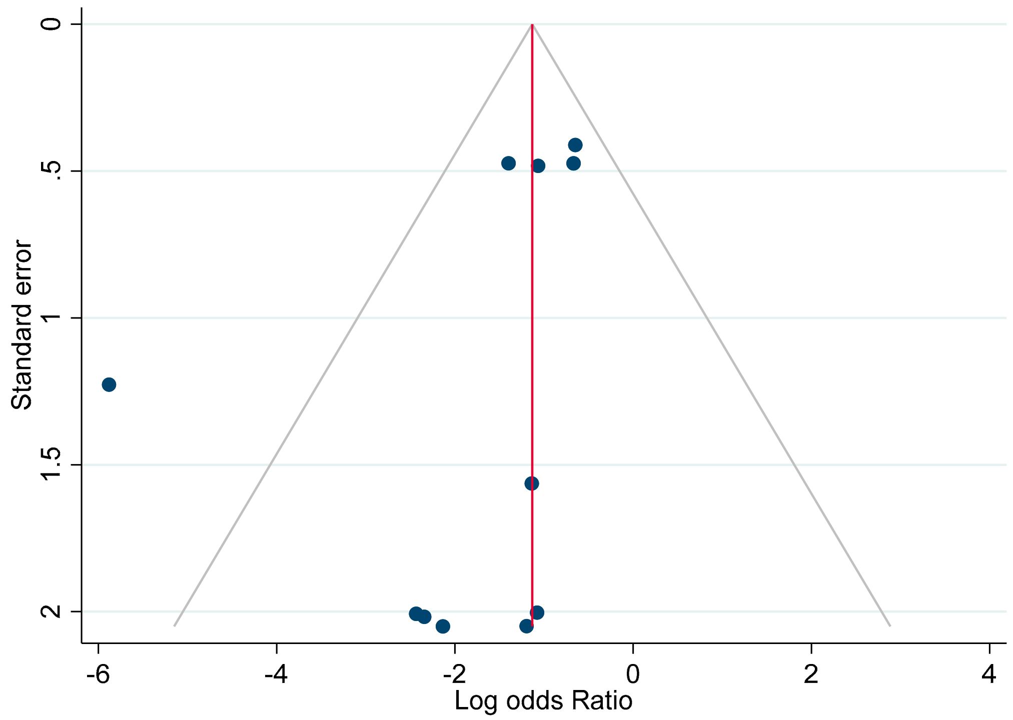

Supplement: Supplementary file 1 [file jcm-11-03063-s001.zip › jcm-1699583-supplementary.pdf]
